# Recurrent Glioblastoma—Molecular Underpinnings and Evolving Treatment Paradigms

**DOI:** 10.3390/ijms25126733

**Published:** 2024-06-19

**Authors:** Christopher Chang, Velina S. Chavarro, Jakob V. E. Gerstl, Sarah E. Blitz, Lennard Spanehl, Daniel Dubinski, Pablo A. Valdes, Lily N. Tran, Saksham Gupta, Luisa Esposito, Debora Mazzetti, Florian A. Gessler, Omar Arnaout, Timothy R. Smith, Gregory K. Friedman, Pierpaolo Peruzzi, Joshua D. Bernstock

**Affiliations:** 1 Warren Alpert Medical School, Brown University, Providence, RI 02912, USA; christopher_chang@brown.edu; 2 Department of Neurosurgery, Brigham and Women’s Hospital, Boston, MA 02115, USA; vchavarro@mgh.harvard.edu (V.S.C.); jgerstl@bwh.harvard.edu (J.V.E.G.); sarahblitz@hms.harvard.edu (S.E.B.); lspanehl@bwh.harvard.edu (L.S.); sgupta@bwh.harvard.edu (S.G.); dmazzetti@bwh.harvard.edu (D.M.); oarnaout@bwh.harvard.edu (O.A.); trsmith@bwh.harvard.edu (T.R.S.); jbernstock@bwh.harvard.edu (J.D.B.); 3 Harvard Medical School, Harvard University, Boston, MA 02115, USA; 4 Department of Neurosurgery, University of Rostock, 18055 Rostock, Germany; daniel.dubinski@med.uni-rostock.de (D.D.); florian.gessler@med.uni-rostock.de (F.A.G.); 5 Department of Neurosurgery, University of Texas Medical Branch, Galveston, TX 77555, USA; paavalde@utmb.edu; 6 Division of Biology and Medicine, Brown University, Providence, RI 02912, USA; lily_n_tran@brown.edu; 7 Department of Medicine and Surgery, Unicamillus University, 00131 Rome, Italy; luisaesposito99@icloud.com; 8 Division of Pediatrics, Neuro-Oncology Section, MD Anderson Cancer Center, Houston, TX 77030, USA; gkfriedman@mdanderson.org; 9 David H. Koch Institute for Integrative Cancer Research, Massachusetts Institute of Technology, Cambridge, MA 02139, USA

**Keywords:** recurrent glioblastoma, neurosurgery, neuro-oncology

## Abstract

Glioblastoma is the most common and lethal central nervous system malignancy with a median survival after progression of only 6–9 months. Major biochemical mechanisms implicated in glioblastoma recurrence include aberrant molecular pathways, a recurrence-inducing tumor microenvironment, and epigenetic modifications. Contemporary standard-of-care (surgery, radiation, chemotherapy, and tumor treating fields) helps to control the primary tumor but rarely prevents relapse. Cytoreductive treatment such as surgery has shown benefits in recurrent glioblastoma; however, its use remains controversial. Several innovative treatments are emerging for recurrent glioblastoma, including checkpoint inhibitors, chimeric antigen receptor T cell therapy, oncolytic virotherapy, nanoparticle delivery, laser interstitial thermal therapy, and photodynamic therapy. This review seeks to provide readers with an overview of (1) recent discoveries in the molecular basis of recurrence; (2) the role of surgery in treating recurrence; and (3) novel treatment paradigms emerging for recurrent glioblastoma.

## 1. Introduction

Glioblastoma is the most common and aggressive primary central nervous system (CNS) tumor. The current standard of care for newly diagnosed glioblastoma includes maximal safe resection followed by radiation therapy and temozolomide (TMZ) [1]. Moreover, regimens adding tumor treating fields (TTFs) are increasingly being considered standard of care [2]. Other variations in treatment protocols include differences depending on age and methylation status of O6-methylguanine-DNA methyltransferase (*MGMT*) [3,4,5]. However, despite well-established protocols for initial treatment and continued efforts to improve treatment options, the median overall survival for newly diagnosed glioblastoma remains poor at 15.6 months after initial treatment, with recurrence typically occurring within 6–9 months of initial diagnosis [6,7]. 

Maximal safe resection represents the cornerstone of current newly diagnosed glioblastoma treatment, demonstrating both a survival benefit and improved quality of life [8]. As such, new techniques have been developed to maximize resections such as intraoperative MRI or fluorescent guiding agents like 5-aminolevulinic acid (5-ALA), which preferentially accumulates in malignant tissues as protoporphyrin IX [9,10,11,12,13]. However, the complete removal of tumor cells is not possible with contemporary surgery as infiltrating glioblastoma cells persist in the brain, extending beyond the visible tumor [14,15,16]. In fact, postmortem studies have demonstrated that neoplastic cells extend beyond visible enhancement on imaging, making even supramaximal surgery non-curative [17]. Furthermore, as a consequence of initial chemoradiotherapy, residual tumor cells may undergo epigenetic modifications and acquire DNA mismatch repair mutations, which can induce resistance to subsequent therapy [18]. The resistance to chemotherapy and radiation and challenges concerning maximal safe resection continue to be core problems in treating recurrent glioblastoma.

Accordingly, this review first aims to summarize current research in recurrent glioblastoma such as the molecular pathways and genetic mutations that drive cell proliferation (*TERT*, *PTEN*, PI3K/Akt, *MSH6*, and *LTBP4*) and paracrine signaling of glioma stem cells (GSCs) [19,20]. Secondly, we discuss the advantages and limitations of resection in recurrent glioblastoma and alternatives including laser interstitial thermal therapy (LITT). Finally, we discuss novel treatment paradigms in recurrent glioblastoma including checkpoint inhibitors, chimeric antigen receptor T cell (CAR-T cell) therapy, and OV therapy.

## 2. Molecular Mechanisms of Recurrence

### 2.1. Genetic Mutations in Newly Diagnosed Glioblastoma and Recurrent Glioblastoma

To understand the genetic and molecular mechanisms of recurrent glioblastoma, the changes from newly diagnosed glioblastoma to recurrent glioblastoma must be considered. Although the total number of genetic mutations is reported to be relatively stable between newly diagnosed and recurrent glioblastoma [16,21], several dominant mutations at initial diagnosis differ compared with recurrence (Table 1). In recurrent glioblastoma, there is the preservation of clonal mutations such as telomerase reverse transcriptase (*TERT*) [22] and phosphatase and tensin homolog (*PTEN*) [23], with a relative loss of epidermal growth factor receptor (*EGFR*) mutations and amplification [21,24,25] and overexpression of platelet-derived growth factor receptor (*PDGF-R*). On the other hand, acquired hypermethylation of mismatch repair genes *MSH6*, *LTBP4*, and *ALKBH5* are almost exclusively observed in recurrent glioblastoma post-initial treatment [21,26].

Gene alterations in *TERT* have also shown relative clonal stability between newly diagnosed glioblastoma and recurrent glioblastoma [21]. The *TERT* gene encodes the catalytic domain of telomerase and maintains telomere length and stability. TERT is normally not expressed in somatic cells but can be activated in various types of cancer, leading to uncontrolled proliferation [27]. In both newly diagnosed glioblastoma and recurrent glioblastoma, the *TERT* gene promoter mutation is commonly conserved, leading to an aggressive, highly proliferative phenotype [53]. Although there is a paucity of information on how *TERT* mutations could impact tumor recurrence, the presence of *TERT* promoter mutations is associated with other biomarkers relevant to tumor cell survival/progression such as *EGFR* amplifications (a dominant mutation in newly diagnosed glioblastoma prior to treatment) and *PTEN* mutations (dominant in recurrent glioblastoma) [54,55,56]. 

*PTEN* is a tumor suppressor gene with a high prevalence of loss in newly diagnosed glioblastoma, sustained at similar levels in recurrent glioblastoma [21,23,34,57,58,59]. *PTEN* catalyzes the hydrolysis of PIP3, which inhibits the activation of the downstream PI3K/Akt pathway, which in turn regulates cell cycle progression [60,61,62]. Thus, the loss of PTEN characteristically results in the loss of PI3K/Akt-dependent cell cycle inhibition, leading to uncontrolled cell proliferation [63]. These characteristics can also be attributed to the flux through the mammalian target of rapamycin (mTOR), a potent downstream effector of the PI3K/Akt that allows for protein translation, cell growth, proliferation, and survival [64]. An in vivo study by Zhang et al. showed that FK506-binding protein 12 (FKBP12) ligand co-administered with mTOR inhibitors enabled the retention and brain-specific action of mTOR inhibitors, leading to suppressed tumor growth and improved survival without on-target side effects [65]. Upstream of mTOR, the PI3K/Akt pathway is one of the major pathways implicated in the survival, proliferation, invasion, and migration of glioblastoma cancer cells [25]. Studies have identified PI3K-activating mutations in 67–82% of newly diagnosed glioblastoma [34,66] and 91% of recurrent glioblastoma [24,34], indicating a highly conserved pathway of cell proliferation [35]. Multiple pre-clinical studies inhibiting PI3K/Akt substrates have yielded positive treatment results in vitro [67,68]. Furthermore, long non-coding RNAs (lncRNAs) upregulate PI3K/Akt signaling [36,69]. The inhibition of lncRNAs such as LINC01426 and the knockdown of long stress-induced non-coding transcript 5 gene (*LSINCT5*) inhibit recurrence by attenuating the migratory ability of GSCs via inhibition of PI3K/Akt signaling pathways [70]. PI3K/Akt is not only directly upregulated in GCS lines but also a significant player in the TME. For example, glioblastoma cell lines can repurpose extracellular vesicles to increase proliferation and cell migration via the upregulation of PI3K/Akt [37], also promoting recurrence.

*EGFR* was the first described transmembrane receptor tyrosine kinase, which results in the activation of downstream pathways regulating proliferation, angiogenesis, migration, survival, and cell differentiation [71,72]. *EGFR* gene amplifications are present in more than half of glioblastoma [73,74], while EGFRvIII, a truncated receptor resulting from the deletion of exons 2 to 7, is the most common *EGFR* oncogenic mutation in glioblastoma [75]. EGFRvIII renders the receptor unable to bind any ligand [30], but it is usually co-expressed with the wild-type *EGFR*. Co-expression results in an autocrine loop that induces glioblastoma cells to produce both the receptor and the ligand [76]. EGFR/EGFRvIII downstream signaling includes constitutive activation of PIP3K/Akt, RAS/MAPK, and Bcl-Xl and has been implicated in glioblastoma cell proliferation, inhibiting apoptosis and enhancing glioblastoma invasiveness [31]. EGFRvIII-specific activation of dedicator of cytokinesis 1 (DOCK1) mediates cell growth and migration [32], whereas EGFRvIII-specific activation of SRC family kinases promotes cell survival in low-energy states [33]. EGFR’s most important ligands in glioblastoma include epidermal growth factor (EGF) and transforming growth factor-alpha (TGF-α) [74].

The overexpression of growth factor receptors plays a significant role in glioblastoma recurrence through glioma stem cell renewal and maintenance. In addition to *EGFR*, *PDGF-R* also serves as an important gene often overexpressed in newly diagnosed and recurrent glioblastoma [77]. In a study by Kim et al., the researchers observed that the high expression of PDGF-R β in GSCs led to increased expression of SOX-2 and decreased glial fibrillary acid protein (GFAP), demonstrating maintenance of a stem cell-like state in GSCs. In further experiments, Kim et al. used fluorescence-activated cell sorting (FACS) and showed that the inhibition of PDGF-R β resulted in decreased proportions of GSCs in the S phase of the cell cycle [38]. These experiments jointly suggest the important role of PDGF-R β signaling in GSC maintenance. Similarly, recent research by Lane et al. determined that the inhibition of PDGF-R α/β resulted in the outgrowth of neurites in glioblastoma and GSC cell lines [39]. The inhibition of PDGF-R serves as a potential therapeutic target to disrupt a persistent stem-cell-like state, deterring recurrence. 

Although there is significant concordance in the genetic makeup between primary and recurrent glioblastomas, several mutational variations have been established between the two. Importantly, recurrent glioblastomas acquire new mutations following initial chemoradiation, including mutations in DNA mismatch repair (MMR) genes *MSH6*, *MLH*, and *LTBP4* [78]. Indraccolo et al. [78] compared the expression of MMR proteins and matched whole exome sequences of newly diagnosed glioblastoma and subsequent recurrent glioblastomas in 57 patients. In 30% of the recurrent glioblastoma samples, a decrease in MMR protein expression was observed, with concurrent total loss, partial loss, and/or loss of function of *MSH2* or *MSH6* gene expression. Wang et al. [21] confirmed these results in 65 matched newly diagnosed glioblastoma and recurrent glioblastoma transcriptome and whole exome samples from six international sites from TCGA. All hypermutated samples were observed in recurrent glioblastoma after treatment with TMZ, with 94% acquiring the following mutations absent in matched newly diagnosed glioblastoma samples: *MSH2* and *MSH6* [79]. Understanding these changes is crucial for targeting recurrent glioblastoma. This acquired resistance to alkylating chemotherapy secondary to MMR *MSH6* gene mutation was rescued by downstream peroxisome proliferator-activated receptor (PPAR) inhibition of DNA repair—a targeted therapy for TMZ-resistant recurrent glioblastoma [79]. Also, recurrent glioblastomas often harbor mutations in the latent TGF-binding protein 4 (*LTBP4*) gene, commonly absent in newly diagnosed glioblastoma-matched samples [21]. The *LTBP4* gene drives the expression of proteins upstream of TGF-β that play a role in glioblastoma cell proliferation and migration. In rare cases, the *BRAF^v600E^* mutation has been implicated in epithelioid glioblastomas [80]; in patients with recurrence harboring such mutations, BRAF inhibitors have shown promise [81].

### 2.2. Epigenetic Modifications in Recurrent Glioblastoma 

**Figure 1:**
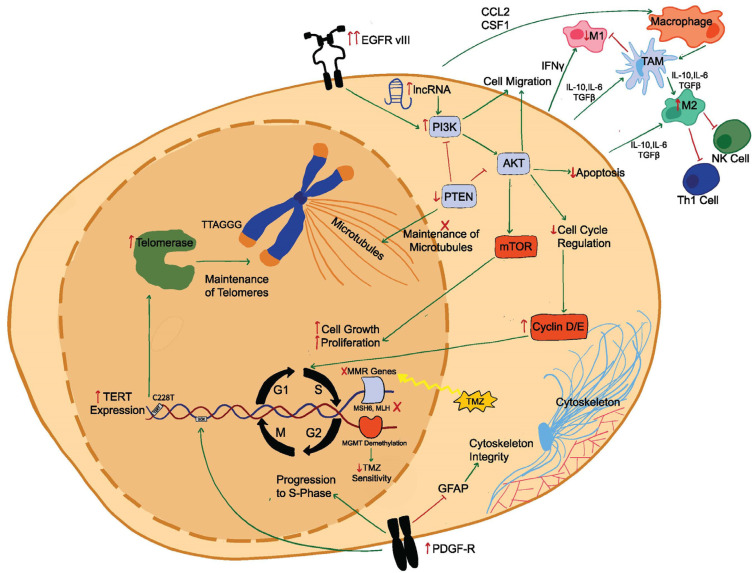
Molecular, epigenetic, and extracellular pathways in recurrent glioblastoma.

Given the high concordance between genetic mutations in newly diagnosed glioblastoma and in recurrent glioblastoma, increasing emphasis is being placed on transcriptional and epigenetic changes as drivers of recurrence [16] (Figure 1). Epigenetic modifications—like DNA methylation and histone acetylation events—can impact tumor behavior through mechanisms like controlling clonal gene expression [82,83]. For example, exposure to paracrine IFN-γ signaling results in epigenetic changes in GSCs [84] and hybrid glioblastoma-tumor-associated macrophage (TAM) cells with anti-inflammatory markers [85,86]. Histone deacetylase 8 (HDAC8) regulates natural killer (NK) cell cytotoxic activity and contributes to a hypoimmunogenic environment [87], while the deletion of ALKBH5 in hypoxic TME results in the recruitment of anti-inflammatory TAMs [20,88]. ALKBH5 is an N^6^-methyladenosine demethylase that results in increased oncogene expression responsible for glioblastoma proliferation. Dong et al. [48] demonstrated that the absence of demethylase activity results in the inhibition of CXCL8 expression (a neutrophil chemoattractant), subsequent IL-8 paracrine signaling, and dampening of pro-inflammatory TAMs. Similarly, Zhang et al. [49] demonstrated that ALKBH5 demethylase activity results in patient-derived glioblastoma cell proliferation, which is suppressed when ALKBH5 is silenced. The tumorigenic effects of ALKBH5 also appear to induce a TMZ-resistant state via the demethylation of SOX2 transcripts, resulting in increased SOX2 expression and Wnt5a/b glioblastoma proliferation signaling.

An extensively studied epigenetic mechanism that regulates disease progression in glioblastoma is MGMT, which encodes the DNA repair protein O^6^-alkylguanine (O^6^-AG) DNA alkyl transferase (AGT) [89]. The MGMT promoter has been an important target in both newly diagnosed glioblastoma and recurrent glioblastoma as the protein product, AGT, yields resistance to alkylating chemotherapy such as TMZ [51,89]. Hypermethylation of MGMT promoter results in silencing and decreased expression of AGT. Consistently, numerous studies and trials have demonstrated a positive effect of MGMT promoter methylation in response to initial treatment with TMZ in newly diagnosed glioblastomas [3,4,5,50]. 

Tyrosine kinase inhibitors can cause GSCs to transition into a slow-cycling, persistent state wherein Notch signaling-dependent developmental programs are upregulated [90]. In the persistent GSC state, H3K27 methyltransferase is downregulated, while H3K27 demethylases of KDM6A and KDM6B are upregulated. KDM6A/B are reported to be essential for persistent GSC survival and formation, as KDM6A/B knockouts demonstrate growth reduction in patient-derived persistent glioblastomas. However, upon transition to the persister state, H3K27 acetylation in GSC is associated with a reduction in H3K27me3 and increases in gene expression near enhancer-like elements. These findings indicate potential therapeutic avenues for persistent GCS that exploit epigenetic shifts in methylation and acetylation patterns [90].

### 2.3. TME Facilitating Glioblastoma Recurrence

Although intrinsic molecular drivers play an important role in tumor behavior, glioblastoma progression is also significantly impacted by the surrounding microenvironment. Although glioblastomas are considered “cold tumors” because of their highly immunosuppressive state, the tumor proper consists of 30–40% non-cancer immune cells [91]. Up to half of these are TAMs—microglia-derived (mgTAM) and monocyte-derived (moTAM) [78,92,93,94]—and the remainder are dendritic cells, T cells, NK cells, and neutrophils [83]. The rate of recurrence and proliferation of glioblastoma has been positively associated with the quantity and phenotype of TAMs, which in turn modulate the activity of T cells, dendritic, and NK cells in the TME [95]. The interaction between GSCs and TAMs in the TME is complex and consists of initial recruitment from the periphery via GSC release of cytokines such as chemokine ligand 2 (CCL2) and colony-stimulating factor 1 (CSF-1) signaling [40,41,42,43]. Once macrophages are within the vicinity of the tumor, they polarize along a pro-inflammatory versus anti-inflammatory axis via a combination of paracrine signals released by GSC and other cells within the TME [96]. IL-10, IL-6, and TGF-β drive polarization of the TME into the anti-inflammatory M2 phenotype, while IFN-γ and STAT1 activity promote a pro-inflammatory M1 phenotype [26,44,95,97,98,99,100].

GSCs and glial cells in the TME release large quantities of TGF-β and IL-10 [44,45,46], enhancing the immunosuppressed environment. Anti-inflammatory TAMs in turn dampen lymphocyte reactivity by inhibiting Th-1 and NK response and infiltration, which enhances Th-2 and Treg activity. This phenomenon is sustained by GSC-inducing genetic changes in TAMs that result in the release of immunosuppressive adenosine [47] and expression of surface molecules that inactivate T cells [83,101]. The cumulative effect is a chronic immunosuppressed state where glioblastoma cells acquire multiple epigenetic modifications and contribute to the hypoimmunogenic TME favorable for survival, proliferation, and resistance to treatment. By understanding and manipulating various components of the TME, glioblastoma treatments can aim to evade immunosuppression to prevent recurrence or treatment resistance.

As neovascularization contributes to maintaining GSCs in the TME, the upregulation of angiogenic elements in glioblastoma has also emerged as an important yet complex player in tumor recurrence and progression. A study in patient-derived glioblastoma cells found that tumor cells supported angiogenic sprouting in hypoxic conditions, forming co-localized networks of sprouting models [102]. One proposed pathway for this occurrence is the upregulation of hypoxia-inducible factor 1 (HIF-1) signaling in the hypoxic TME, releasing proangiogenic growth factors VEGF and basic fibroblast growth factor (bFGF) [103,104]. On the other hand, the upregulation of histamine signaling by GSCs in the TME has also been shown to trigger an increase in the H_1_-Ca^2+^-NF-kB axis independent of VEGF activation. In vivo studies with glioblastoma xenograft mice models revealed that pharmacological blockage of H_1_R using antihistamines impeded the growth of glioblastoma, establishing a potential therapeutic target in the TME for glioblastoma [105]. While it has been well established that neovascularization enables nutrient transport to the TME to promote GSC maintenance, leading to recurrence, the various pathways that directly link angiogenesis to tumor recurrence warrant further investigation. 

## 3. The Role of Surgery in Recurrent Glioblastoma

Glioblastoma cells invade the brain parenchyma diffusely, making complete resection of tumor cells impossible [106,107,108]. Reoperation may be an option for select patients based on recurrence location, preoperative estimate of maximal safe resection of T1 contrast-enhancing disease, preoperative functional status and symptoms, and careful weighing of the risks and benefits [109,110,111,112]. Good candidates for repeat surgery include young patients, Karnofsky Performance Score (KPS) > 70, few comorbidities, and non-eloquent tumor location [113]. However, even in patients who qualify for surgery, the determination of safe and efficacious resection borders often poses an additional challenge because of unspecific FLAIR signals and the limitations of imaging [114]. Although some retrospective studies have reported beneficial outcomes after reoperation, there is no consensus regarding the role of surgery in recurrent glioblastoma [115,116,117]. As such, reoperation in recurrent glioblastoma remains a decision made on a case-by-case basis with multidisciplinary discussions among neurosurgeons, neurooncologists, radiation oncologists, and patients [118]. 

Multiple retrospective studies suggest a surgical benefit in overall survival (OS) in patients with recurrent glioblastoma. Djamel-Eddine et al. [119] assessed outcomes in 2005–2014 and reported significant survival benefits of repeat surgery; the median OS for patients undergoing one, two, and three surgical procedures were 11, 16, and 18 months respectively. However, while adjuvant treatment after the first surgery was standardized using the temozolomide and radiotherapy protocol [1], there was no standard protocol for subsequent resections, significantly confounding the conclusions regarding the benefit of reoperation on OS in patients with recurrent glioblastoma. In addition, key confounding variables including MGMT methylation status were not accounted for [119]. In another study of 97 patients who underwent reoperation for recurrent glioblastoma, Yong et al. [120] reported an improved median OS of 12.4 months after surgery for recurrent glioblastoma. This effect, though, was confounded by variance in post-operative residual tumor size. Similar to the work by Djamel-Eddine et al. [119], patients in this cohort who underwent repeat surgery were also carefully selected, which introduced selection bias. Nevertheless, a larger retrospective cohort of 503 patients, allowing for multivariate analysis, by Ringel et al. [107] demonstrated the prognostic benefit of surgery in recurrent glioblastoma. Finally, a recent study by Karschnia et al. [121] investigated the prognostic value of surgery at recurrence in 681 patients with recurrent glioblastoma. The study found a survival benefit in patients undergoing resection (11 months), compared with those that did not (7 months), after stratifying for potential clinical confounders [121].

On the other hand, numerous studies have pointed to the lack of benefit of repeat surgery for recurrent glioblastoma. González et al. [112] completed a 15-year retrospective analysis of 350 patients with recurrent glioblastoma. The authors stratified patients eligible for repeat surgery into one of two groups as follows: (1) those who underwent reoperation and adjuvant chemotherapy and (2) those treated only with chemotherapy. Patients who underwent reoperation with adjuvant chemotherapy had a longer median OS of 25 months vs. 17 months compared with the non-surgical arm. Univariate analysis revealed that six cycles of TMZ treatment as well as reoperation impacted OS; however, statistical significance was absent when the most significant covariates were adjusted for [112]. These results were confirmed by Franceschi et al. in a cohort of 232 patients with recurrent glioblastoma [116]. Despite the prolonged median OS in patients who underwent surgery (25.8 vs. 18.6 months), MGMT methylation, age, and progression-free survival (PFS) at 6 months were more strongly correlated with OS than secondary surgery. Nava et al. [111] reported similar findings among 764 patients with recurrent glioblastoma. Their results demonstrated that radiotherapy with temozolomide was the strongest predictor of OS and PFS in recurrent glioblastoma, while reoperation had a minimal effect [111]. Despite conflicting results, most clinicians agree that there is a benefit for reoperation in carefully selected patients [122,123,124,125]. 

Several factors are important to consider when performing a reoperation on recurrent glioblastoma including the functional status of the patient (i.e., KPS), mass effect, possible preservation of functional tissue (e.g., motor pathways, language pathways), and, crucially, the anticipated extent of resection. Importantly, Suchorska et al. reported that incomplete tumor resection was associated with worse post-recurrence survival compared with no surgery (6.5 months vs. 9.8 months) [126]. Bloch et al. [115] corroborated these findings in a cohort of 107 patients, defining gross-total resection (GTR) as the removal of 95% or greater of tumor volume and subtotal resection (STR) as the removal of less than or equal to 95% of tumor volume. Patients who underwent GTR at recurrence had a significantly greater median survival of 19.0 months compared with those who underwent STR with a median survival of 15.9 months [115]. Of note, defining the extent of resection (EOR) is critical for understanding the relationship between the amount of tumor removed and associated survival benefit. Oppenlander et al. [110] performed a study on 170 patients with recurrent glioblastoma and found a significant survival advantage with as little as 80% EOR, with additional recursive partitioning analysis demonstrating a continued benefit to OS with the highest levels of EOR. Karschnia et al. [121] reported that a residual contrast-enhancing volume threshold ≤1 cm^3^ compared with >1 cm^3^ was associated with increased survival. Furthermore, their study also found an exponential increase in hazard ratios for death with higher residual contrast-enhancing tumors. Future studies are required to investigate survival outcomes based on different amounts of tumor resection to discriminate the threshold at which patients begin to experience a survival benefit after reoperation for recurrent glioblastoma.

Patient age is another significant factor when considering reoperation for recurrent glioblastoma. Woernle et al. [117] performed a retrospective analysis of 98 patients who underwent initial surgical resection followed by radiotherapy with concomitant TMZ. The authors reported that repeat surgery resulted in improved OS in the younger group. Neville et al. [127] reported similar findings in a group of 286 patients who had adjuvant carmustine or TMZ. OS at 6, 12, and 24 months was greater for patients who also underwent reoperation for recurrent glioblastoma [127]. However, OS was confounded by younger age, higher KPS, the type of initial management, and reoperation extent. As previously mentioned, a major limitation of these studies is selection bias—patients who undergo repeat surgery are typically younger and otherwise healthier. Furthermore, age influences multiple important prognostic molecular markers such as *TERT* mutation and response to chemotherapy. 

Fluorescence-guided surgery (FGS) using 5-ALA represents a recent promising development in the surgical resection of glioblastoma. FGS resulted in double the rates of GTR in newly diagnosed glioblastoma in a first phase III trial evaluating FGS for brain tumors (65% fluorescence group vs. 36% white light group) [9]. The pro-drug 5-ALA is administered orally to patients just prior to surgery, which leads to the accumulation of a red fluorescent agent, protoporphyrin IX (PpIX). The use of intraoperative microscopes modified for fluorescence imaging, including a violet excitation light and long pass filters to collect the emitted red fluorescence from PpIX, enables neurosurgeons to visualize the PpIX that accumulates in tissues [128,129]. In addition, 5-ALA has been used in recurrent glioblastoma to aid in the visualization of tumor tissue [129,130,131,132,133,134,135]. For example, Hickman et al. analyzed 63 recurrent glioblastoma tumors, of which 84.1% (53/63) showed visible PpIX fluorescence, with most non-fluorescent tumors being isocitrate dehydrogenase (IDH) mutants [136]. Although 5-ALA FGS is considered safe, PpIX fluorescence can be found in reactive-only tissue without the presence of tumor cells in recurrent glioblastoma [136,137]. Accordingly, surgeons should be aware of such false positives, as one study found that 13 out of 313 patients with recurrent glioblastoma demonstrated positive 5-ALA-PpIX fluorescence in reactive-only tissue. Although 5-ALA-PpIX FGS can serve as a powerful adjunct in recurrent glioblastoma cases to help surgeons maximize the extent of resection, like every tool, the surgeon should know the limitations of the technology [137].

The surgical decision to reoperate is heavily guided by factors such as patient age, KPS scores, comorbidities, tumor location, and pre-operative estimation of expected residual contrast enhancement (i.e., ideally < 1 cm^3^) meanwhile limiting post-operative deficits. Despite the various nuances in determining the efficacy and outcomes of reoperation, most studies showed that younger patients and those with fewer comorbidities have the most favorable reoperation outcomes.

## 4. Evolving Treatment Paradigms for Recurrent Glioblastoma

Current guidelines for recurrent glioblastoma management recommend enrollment in a clinical trial [138] and follow-up with MRI at 3- to 6-month intervals up to 2 years and then every 6 to 12 months up to the 5-year mark [132]. This section discusses evolving treatment paradigms in recurrent glioblastoma, including immune checkpoint inhibition, cellular therapies, oncolytic viral therapy, novel delivery methods, LITT, photodynamic therapy, and intratumoral microdevices.

### 4.1. Checkpoint Inhibitors 

Several immune checkpoint inhibitors targeting T cell-mediated immunity have shown promise in non-CNS solid tumors. The most relevant T cell checkpoints are the programmed cell death 1 (PD-1)/programmed death-ligand 1 (PD-L1) as well as the cytotoxic T-lymphocyte-associated (CTLA) protein axes [139]. These pathways are exploited by glioblastoma tumors to dampen T cell activation, cytotoxicity, and proliferation [140].

Nivolumab and pembrolizumab are two PD-1 receptor blockers that prevent tumor interaction with PD-L1, a protein that inactivates T lymphocytes (Table 2 and Table 3). Cloughesy et al. 2019 and Schalper et al. 2019 demonstrated an OS benefit and increased T-cell receptors among tumor-infiltrating T-lymphocytes, respectively, following nivolumab administration [141,142]. However, in the CheckMate 143 phase 3 trial, published the following year, nivolumab monotherapy compared to bevacizumab in recurrent glioblastoma revealed no improvement in OS [143]. Similarly, the combination of nivolumab and pembrolizumab with/out concurrent bevacizumab yielded no survival benefit [143,144]. Interestingly, in a phase I clinical trial of combination therapy with nivolumab and a regulatable interleukin-12 gene therapy, veledimex, Chiocca et al. [145] demonstrated an increased OS for the combination therapy arm (16.9 months vs. 9.8 months), mediated by increased IFN-γ. Similarly, Lee et al. [146] found that neoadjuvant PD-1 inhibition leads to enhanced IFN-γ signaling and upregulation of CTLA-4, shifting the TME from anti-inflammatory to pro-inflammatory, recruiting cytotoxic T cells. A trial by Nassiri et al. published in *Nature Medicine* in 2023 combined the oncolytic virus DNX-2401 and intravenous pembrolizumab, demonstrating encouraging results in recurrent glioblastoma therapy. In this trial, 56.2% of the patients had stabilized or improved disease, with a median overall survival of 12.5 months. Three patients in the treatment arm had a durable response, remaining alive at 45, 48, and 60 months [147]. 

While trials such as CheckMate 143 show a statistically non-significant survival benefit [143], checkpoint inhibitors target a well-described mechanism in recurrent glioblastoma and still present as a promising combination therapy option for recurrent glioblastoma. Clinical trials investigating the concurrent CTLA-4 and anti-PD-1 treatment of recurrent glioblastoma (NCT04606316) are currently ongoing. 

### 4.2. CAR-T Cell Therapy

Chimeric antigen receptors (CARs) are formed through genetic engineering of T cells against specific tumor antigens (Table 4). The unique benefit of CAR-T cell therapy is T cells’ ability to bypass antigen presentation and required co-stimulatory activation, avoiding tumor-specific ignorance [150]. A variety of CAR-T cell therapies have emerged with targets including EGFR/EGFRvIII, IL-13Rα2, and B7-H3 [151]. *EGFR/EGFRvIII* mutations contribute to increased proliferation, decreased apoptosis, and tumor cell survival in 30–50% of recurrent tumor glioblastoma [76,152]. Despite promising preclinical studies, clinical trials of EGFRvIII-specific CAR-T cell therapy have demonstrated limited efficacy [153,154,155]; however, there have been some favorable reports. A case study by Durgin et al. [156] reported 36-month survival after CAR-T cell EGFRvIII therapy for recurrent glioblastoma. At 29 months, a follow-up MRI brain demonstrated decreased tumor enhancement with persistent peripheral T cell circulation. A similar result was reported by O’Rourke et al. [153], where peripheral expansion of EGFRvIII CAR-T cells resulted in a drop in tumor antigen among five of seven patients who had EGFRvIII CAR-T cell infusions in recurrent glioblastoma enhancing cavities. Additionally, the combination of EGFRvIII CAR-T cells with the immune checkpoint inhibitor pembrolizumab has been demonstrated to be safe, yet not efficacious [157]. An alternative target of CAR-T cell therapy is the IL-13Rα2, expressed almost universally in recurrent glioblastoma. It is a decoy receptor that binds IL-13, inhibiting an immune response [158,159]. IL-13Ra2 is associated with the activation of the PI3K/Akt pathway which mediates tumor progression in recurrent glioblastoma [159,160,161]. Brown et al. recently published phase 1 results using IL-13Rα2 targeting CAR-T cells in 65 patients [162]. An OS of 7.7 months from the time of recurrence was reported; one arm using a refined manufacturing platform delivering IL-13Rα2 targeting CAR-T cells both intraventricularly and intratumorally demonstrated a superior OS of 10.2 months. Finally, interim results from two CAR-T constructs targeting two antigens (EGFR and EGFRvIII by Choi et al. [163] and EGFR and IL-13Rα2 by Bagley et al. [164]) demonstrated rapid tumor regression within days and an acceptable safety profile. 

### 4.3. NK Cell Therapy

NK cell therapies have emerged as a novel treatment serving as an alternative to CART-T therapy [165] (Table 5). NK cells are part of the innate immune system, allowing them to eliminate aberrant cells independent of the T cell activation signal and prevent CAR antigen loss-induced immune escape [166]. Murakami et al. [167] showed that in glioblastoma tumors expressing EGFRvIII growth factors, NK cell lines (CAR-KHYG-1) specific for EGFRvIII could effectively inhibit glioblastoma cell growth via apoptosis. Furthermore, Zhang et al. reported complete lysis of all the ErbB2 cell lines of LN-319, LNT-229, and LN-428 cells by NK-92/5.28.z cells. In in vivo mouse studies, potent antitumor activity and symptom-free survival were demonstrated following stereotactic injection of NK-92/5.28.z. In addition, local therapies of NK cells resulted in robust immune responses curing transplanted syngeneic glioblastoma in four/give immunocompetent mice carrying subcutaneous tumors and five/eight mice carrying intracranial tumors. Impressively, the NK cell injections also led to tumor-specific immunity and memory (IgG titers) after reinjection of glioblastoma cells (ERbB2/GL261) into distal sites, indicating longer-term protection against tumors [168]. With encouraging results in these preclinical studies, NK cell therapy trials are now underway [165].

### 4.4. Oncolytic Virotherapy

OVs utilize viruses to infect tumor cells resulting in cell lysis and release of tumor antigens, activating an immune response [169] (Table 6 and Table 7). Direct intratumoral delivery of OVs allows for bypassing of the BBB [170]. In a phase 2 clinical trial, Todo et al. tested a triple mutated, third-generation oncolytic HSV type 1 (HSV1, G47∆) in 19 adult patients with recurrent glioblastoma [171]. OV was administered intratumorally in six doses, which resulted in increased tumor-infiltrating CD4+/CD8+ lymphocytes on biopsy, suggesting a targeted immune response against glioblastoma tumor antigens [171]. The patient median OS was 20.2 months after OV therapy and 28.8 months after initial surgical resection, leading to the approval of G47∆ in Japan [171,172].

In a phase I trial, Friedman et al. demonstrated that stereotactic intratumor infusions of a genetically engineered HSV-1, G207 were safe and led to an OS of 12.2 months among 12 children with recurrent high-grade glioma [176]. Evaluation of the TME on biopsy pre- and post-treatment suggested a strong pro-inflammatory T cell response with clusters of T cells sustained between 2 and 9 months post-treatment. Moreover, there was increased T cell infiltration in areas distant from inoculation [176]. More recently, Ling et al. demonstrated that treatment with the oHSV CAN-33110 was associated with changes in tumor T cell counts and clonal diversity, peripheral expansion/contraction of certain T cell clonotypes, and unique tumor transcriptomic signatures of immune activation in a phase 1 trial in recurrent high-grade gliomas [173].

OV combination therapy is of increasing research interest [187]. Nassiri et al. [147] tested a combination therapy of the oncolytic adenovirus DNX-2401 with a checkpoint inhibitor in a cohort of 49 patients with recurrent glioblastoma. A single-dose intratumor delivery of DNX-2401 was followed by intravenous pembrolizumab. The median OS was 12.5 months, greater than the OS after previously reported monotherapies of DNX-2401 (OS: 9.3 months) or PD-1 inhibitors (OS: 9.8 months) [143,181]. Patients showed increased density of immune cells—microglia, macrophages, and CD3+, CD4+, or CD8+ T cells—infiltrating the TME [147]. More importantly, two patients who completed 6 months of pembrolizumab after OV inoculation had an 80% tumor volume reduction and remained alive without progression at the time of publication. 

### 4.5. Novel Delivery Methods 

Failure to prevent and treat glioblastoma recurrence may partially be attributed to challenges of drug delivery through the BBB [188]. Novel delivery approaches that couple effective delivery protocols with carriers, including intraventricular infusion of OV and intranasal nanoparticle delivery, show promise in overcoming the delivery challenge set by the BBB [147,171,176,189]. These treatments enable a more focal treatment of glioblastoma, limiting systemic side effects and directly preventing recurrence [190].

Two major disadvantages of intravenous drug administration are the systemic toxicities and the inability of molecules to bypass the BBB. Local intratumor delivery has been the main strategy for overcoming this challenge. However, each treatment infusion requires an invasive neurosurgical procedure, leading to increased complications due to repeat surgeries [191]. Intrathecal or intraventricular delivery is an alternative that could overcome the BBB. This approach has historically been avoided as it was considered high-risk CNS toxicity. In a pre-clinical study, Kang et al. [192] demonstrated that intraventricular delivery of OV G207 in a murine model of CNS disease resulted in increased endothelial-cell mediated toxicity, which could be rescued with a pre-treatment of low-dose OV G207 [191,193].

Studies are also investigating technologies that can increase the CNS concentration of systemically administered drugs. For example, MR-guided ultrasound (MRgFUS) results in the oscillation of pre-formed IV-infused microbubbles, which concentrate the acoustic energy of ultrasound. This causes a transient mechanical separation of endothelial cells, thus “opening” the BBB [194]. Abrahao et al. [195] tested the safety of MRgFUS in a first-in-human feasibility study among four patients with amyotrophic lateral sclerosis. Increased transient BBB permeability, assessed with gadolinium leakage, was achieved in all patients. Multiple pre-clinical glioma models have demonstrated drug delivery safety using MRgFUS [196]. More recently, focal delivery of CAR-T cell therapy, which otherwise results in a potent systemic inflammatory effect, has demonstrated the precision of drug delivery to the local TME when combined with MRgFUS [197].

Nanocarriers and nanotechnology-based drug delivery have also demonstrated positive results in preclinical studies by effectively transporting drugs into the CNS. Because of their small size, nanoparticles can penetrate small capillaries and efficiently pass the BBB through receptor-mediated endocytosis [198,199]. Nanoparticles utilized in glioblastoma treatment have chemotherapeutic agents trapped inside their matrix. The external core membrane undergoes biodegradation within the CNS [200]. Preclinical studies have reported that nanoparticle-delivering agents that inhibit lactate metabolism resulted in the halting of cancer cell growth [201]. The nanoparticles contained lactate oxidase, which, in the hypoxic TME, converted lactate to pyruvic acid and hydrogen peroxide, blocking histone expression and inducing cell cycle arrest. Kumthekar et al. [202] demonstrated the feasibility of using nanoparticles to deliver small interfering RNA (siRNA) in glioblastoma tumors of non-human primates and in a human phase 0 clinical trial. They reported effective localization of gold nanoparticles in tumor cells. Another study investigated intranasally administered polymeric nanoparticles delivering bevacizumab, taking advantage of this technology and a novel administration route [189]. In the preclinical study by Sousa et al. [189], the authors reported that an intranasal administration of nanoparticles led to direct delivery to the CNS, bypassing the BBB through the olfactory and trigeminal nerves [203]. Their results showed that intranasal administration improved CNS bioavailability of bevacizumab, decreasing systemic side effects. However, the therapy did not result in significant tumor shrinkage when comparing nanoparticle delivery to the intravenous administration arms. This, though, is likely explained by the effect of bevacizumab rather than the delivery method [189].

### 4.6. Laser Interstitial Thermal Therapy, Electrical Fields, Photodynamic Therapy, and Intratumoral Microdevices

LITT presents a potential adjunct to resective surgery [204]. LITT uses an optical fiber that generates heat, leading to tumor cell necrosis or apoptosis [190,204]. Moreover, LITT can sensitize glioma cells to other treatment modalities by altering this primed TME; it can cause immune upregulation and BBB disruption [205,206,207,208]. Mohammadi et al. [204] showed a correlation between tissue damage in recurrent glioblastoma regions with LITT and favorable PFS. Reoperation requires careful patient selection and has high complication rates, to which LITT presents an alternative with comparable outcomes [209,210].

Even less invasive, the Optune TTF device was approved by the FDA in 2011 as a treatment for recurrent glioblastoma [211]. The TTF electrodes are applied to the patient’s scalp and deliver low-intensity and intermediate-frequency alternating electric fields [2,212]. The mechanism is thought to interfere with the polymerization and depolymerization of microtubules in mitotic spindles, leading to disruption in cell replication and cell death [212]. Dono et al. suggested that the aberrant progression into mitosis because of *PTEN* mutations in recurrent glioblastoma is halted by TTF through the mechanism of mitotic arrest/delay, leading to improved median survival in recurrent glioblastoma patients with *PTEN* mutations [213]. An established phase 3 clinical trial by Stupp and collaborators showed that TTF devices when worn 18–24 h a day led to similar OS in patients with recurrent glioblastoma undergoing chemotherapy [214]. Shortly after the FDA approval of Optune, Mrugala et al. performed an analysis of the clinical outcomes of TTF device utilization in patients with recurrent glioblastoma across 91 sites in the U.S. Their results revealed that increased compliance with wearing the device (daily compliance ≥ 75%, or ≥18 h daily) led to significantly better median OS at 13.5 months vs. 4.0 months compared with patients who were not compliant [215]. Dono et al. reported that within the TTF monotherapy group, TTF improved the median PPS of recurrent glioblastoma patients with *PTEN* mutations, yet this improvement did not reach statistical significance (13.9 months versus 10.9 months, *p* = 0.068) [213]. Despite questions on patient selection and lack of multivariate analyses, the largest benefits of TTF include a minimally invasive approach and an acceptable safety profile [2,211,215,216]. Unfortunately, despite these promising results, uptake in standard-of-care treatment plans has been slow [2,217].

Another potential treatment for recurrent glioblastoma is photodynamic therapy (PDT) [218]. In PDT, a photosensitizer is activated using a specific wavelength of light, which leads to the generation of reactive oxygen species in tissue that has accumulated the photosensitizer and undergone irradiation, which ultimately leads to the death of tumor cells [219]. Agents that have been used clinically for PDT in gliomas include Photofrin, Temoporfin, HpD, and 5-aminolevulinic acid, which leads to overproduction of the photosensitizer protoporphyrin IX [220,221,222,223]. For example, a study in Japan used the photosensitizer talaporfin sodium for PDT in recurrent glioblastoma, where the authors analyzed a group of 70 patients undergoing surgery plus PDT and 38 control patients undergoing surgery only. The PDT group had improved PFS and OS compared to the surgery-only group (5.7 vs. 2.2 months PFS and 16 vs. 12.8 months OS, respectively), and the effect of PDT on OS was maintained after univariate and multivariate analyses. Despite numerous studies with encouraging results, PDT for glioblastoma has not yielded a successful phase III trial. However, there is active research in new photosensitizers for PDT, delivery methods, and ongoing clinical trials using PDT on glioblastoma, including a trial on recurrent glioblastoma using 5-aminlevulinic acid [224].

New technologies can also be used to guide treatment strategies. Recently, in a first-in-human clinical trial, Peruzzi et al. [225] demonstrated the safety and efficacy of a novel biocompatible intratumoral microdevice (IMD) used to deliver nano-doses of chemotherapy to different areas of the TME in real time during tumor resection. The IMDs are inserted into the tumor at the beginning of surgery and are removed at the end of surgery. This approach acknowledges the large heterogeneity in glioblastoma and non-tumor cells within the TME and samples the sensitivity of the tumor to different chemotherapy agents. Besides demonstrating safety and an excellent adverse event profile associated with the IMD, Peruzzi et al. [225] reported that a patient with recurrent glioblastoma had a tumor resistant to all tested chemotherapeutic agents and avoided ineffective adjuvant chemotherapy and unnecessary potential systemic toxicities from the chemotherapy. Sampling chemotherapy sensitivity in an individual patient’s TME in real time demonstrates the potential to personalize adjuvant therapy. Such personalized therapeutic approaches may become critical as large molecular and genetic data have not added practical therapeutic value because of the multiplicity of involved pathways and the epigenetic modifications tumor cells undergo after initial therapy.

## 5. Conclusions

Glioblastoma is the most aggressive malignancy of the CNS and, despite standard-of-care resection and chemoradiation, most patients succumb to the disease. Extensive research during the past decade has uncovered molecular and genetic bases of glioblastoma recurrence, including driver mutations and major molecular pathways that sustain tumor cell proliferation. There has also been a perspective shift to investigate epigenetic modifications of recurrent tumors and a focus on understanding the interactions between tumor cells and non-tumor cells in the TME. A greater understanding of these mechanisms has guided advancements in treatments. For example, immune checkpoint inhibitors and engineered CAR-T cells utilize lymphocytes’ ability to create a local pro-inflammatory state unfavorable for tumor cell growth and invasion. On the other hand, oncolytic virotherapy introduces a viral pathogen targeted to the tumor cavity to activate a pro-inflammatory response. Novel drug delivery approaches, such as viral capsids or nanoparticles, can also help overcome treatment barriers, like bypassing the BBB and avoiding systemic side effects. Additionally, traditional surgery still plays a critical cytoreductive role in prolonging OS for select patients, although reoperation is often limited by patient characteristics such as age, performance status, tumor location, and involvement of critical brain regions. Alternative or additive treatments include LITT and TTFs. There is still more work required to identify effective treatments and treatment combinations for recurrent glioblastoma, but ongoing research into the mechanisms of recurrence and treatment evasion will help guide future trials.

## Figures and Tables

**Table 1:** Molecular, epigenetic, and paracrine signaling in recurrent glioblastoma.

Gene, Epigenetic Modification, or Paracrine Signaling	Mutational Characteristics in Recurrent Glioblastoma	References
TERT	Highly conserved mutation with the *TERT* promoter as the most conserved mutation Correlated with *EGFR* amplifications, leading to tumor survival and progression	[27]
	*TERT* C228T mutation confers poorer prognosis than C250T	[28]
	PTEN aberrations lead to microtubule disruptions, resulting in improper cell cycle regulation and mitosis	[29]
EGFR	EGFRvIII results in truncated receptor co-expressed with the wtEGFR, which leads to constitutively activated downstream EGFR signaling EGFRvIII specific signaling includes DOCK1 and SFK pathways	[30,31,32,33]
PI3K/Akt	PI3K-activating mutations have been found in 91% of recurrent glioblastomas	[34]
	Unregulated cell cycle progression	[34,35]
	lncRNA upregulates PI3K and leads to stem cell migration	[21,36]
	Glioblastoma-derived EVs upregulate PI3K/Akt proliferation and migratory signaling	[37]
PDGF-R α/β	High expression of PDGF-R β maintains GSC renewal and survival	[38]
	Knockdown of PDGF-R α/β promotes neurites differentiation in GSC and glioblastoma cell lines	[39]
Paracrine signaling via macrophages	CL2 and CSF1 recruit peripheral macrophages	[40,41,42,43]
	IL-10 and TGF-β signaling polarize TAMs to anti-inflammatory M2	[44,45,46]
	M2 inhibit Th-1 and NK cell responses	[47]
Epigenetic modifications	*MSH6*, *MLH*, and *LTBP4* lead to resistance to TMZ	[21,26]
	*LTBP4* mutations lead IDH upregulation and increased cell proliferation	[21]
	ALKBH5 inhibits CXCL8 in hypoxic TME, leading to GSC proliferation	[48,49]
	Demethylation of MGMT conferred to decreased TMZ sensitivity and shorter survival	[50]
	MGMT promoter methylation is decreased in recurrent glioblastoma when compared with newly diagnosed glioblastoma	[51,52]

TERT = telomerase reverse transcriptase; EGFR = epidermal growth factor receptor; PTEN = phosphatase and tensin homolog; EGFRvIII = mutated EGFR; DOCK1 = dedicator of cytokinesis 1; SFK = SRC family kinases; PI3K = phosphoinositide 3 kinase; lncRNA = long non-coding RNA; EVs = extracellular vesicles; PDGF-R = platelet-derived growth factor receptor; GSC = glioblastoma stem cell; CSF1 = colony-stimulating factor 1; TGF-β = transforming growth factor β; TAM = tumor-associated macrophage; LTBP4 = latent transforming growth factor beta binding protein 4; IDH = isocitrate dehydrogenase; TME = tumor microenvironment; TMZ = temozolomide; MGMT = O(6)-methylguanine-DNA-methyltransferase.

**Table 2:** Published phase 2 and 3 immune checkpoint inhibitor trials in recurrent glioblastoma.

Author/Trial Name/Identifier	Agent/Modality	Study Phase	Study Outcome
Reardon et al., 2020 [143], CheckMate-143, NCT02017717	Nivolumab vs. bevacizumab	Phase 3	mPFS = 1.5 months (nivolumab) vs. 3.5 months (bevacizumab); mOS = 9.8 months (nivolumab) vs. 10.0 months (bevacizumab).
Nayak et al., 2021 [148], NCT02337491	Pembrolizumab + bevacizumab vs. pembrolizumab monotherapy	Phase 2	PFS-6 = 26.0% (pembrolizumab + bevacizumab) vs. 6.7% (pembrolizumab monotherapy); mOS = 8.8 months (pembrolizumab + bevacizumab) vs. 10.3 months (pembrolizumab monotherapy); ORR = 20% (pembrolizumab + bevacizumab) vs. 0% (pembrolizumab monotherapy).
Cloughesy et al., 2019 [141]	Pembrolizumab (neoadjuvant + adjuvant) vs. pembrolizumab (adjuvant only)	Phase 2	mPFS = 3.3 months (neoadjuvant + adjuvant) vs. 2.4 months (adjuvant only); mOS = 13.7 months (neoadjuvant + adjuvant) vs. 7.5 months (adjuvant only).
Schalper et al., 2019 [142], NCT02550249	Neoadjuvant nivolumab (single arm)	Phase 2	mPFS = 4.1 months; mOS = 7.3 months; Higher immune cell infiltration and TCR diversity.
Reardon et al., 2018 [149], NCT02335918	Nivolumab + varlilumab	Phase 2	mOS = 9.7 months.

mPFS = median progression-free survival; mOS = median overall survival; PFS-6 = 6-month progression free survival; ORR = objective response rate; TCR = T cell receptor.

**Table 3:** Ongoing immune checkpoint inhibitor trials in recurrent glioblastoma.

Author/Trial Name/Identifier	Status	Agent/Modality	Primary Objective	Study Phase
NCT04145115	Recruiting	Ipilimumab + nivolumab	Tumor response by modified RANO in recurrent glioblastoma with high mutational burden.	Phase 2
NCT04323046	Active, not recruiting	Ipilimumab + nivolumab	TME changes following neoadjuvant nivolumab and placebo, ipilimumab and placebo, and nivolumab and ipilimumab. Safety and tolerability of neoadjuvant nivolumab and placebo, ipilimumab and placebo, and nivolumab and ipilimumab.	Phase 1
NCT03890952	Active, not recruiting	Nivolumab + bevacizumab (BEV)	Number of indels as determined using mRNA sequencing.	Phase 2
NCT04201873	Recruiting	Pembrolizumab + ATL-DC vaccine	Influence of pembrolizumab on cell cycle-related genetic signatures within the TME. Influence of ATL-DC vaccination on peripheral T cell response. Safety/tolerability of pembrolizumab and ATL-DC vaccination.	Phase 1
NCT04013672	Active, not recruiting	SurVaxM + Sargramostim + Montanide ISA 51	PFS-6	Phase 2
NCT04013672	Active, not recruiting	SurVaxM + Sargramostim + Montanide ISA 51	PFS-6	Phase 2
NCT05465954	Recruiting	Pembrolizumab + efineptakin alfa	OS-9	Phase 2
NCT05465954	Recruiting	Pembrolizumab + efineptakin alfa	OS-9	Phase 2
NCT04479241	Active, not recruiting	Pembrolizumab + lerapolturev	ORR; DOR; DRR	Phase 2
NCT05053880	Recruiting	Pembrolizumab + ACT001	Adverse events; PFS-6	Phase 1b/2
NCT05463848	Recruiting	Pembrolizumab + Olaparib + Temozolomide	TIL density; PFS-6	Phase 2

RANO = Response Assessment in Neuro-Oncology; TME = tumor microenvironment; ATL-DC = adjuvant autologous tumor lysate dendritic cell; PFS-6 = 6-month progression-free survival; OS-9 = 9-month overall survival; ORR = objective response rate; DOR = duration of response; DRR = durable radiographic response; TIL = tumor-infiltrating lymphocyte.

**Table 4:** Ongoing CAR-T cell trials in recurrent glioblastoma.

**Trial Identifier**	**Status**	**Agent/Modality**	**Study Phase**	**Treatment Approach**
NCT01454596	Completed	EGFRvIII CAR transduced PBL	Phase 3	Infusion
NCT00730613	Completed	Autologous lymphocytes	Phase 1	Leukapheresis + infusion
NCT01109095	Completed	HER-2 CAR CMV-specific CTLs	Phase 1	Infusion
NCT02208362	Active, not recruiting	IL13Ralpha2-specific Hinge-optimized 41BB co-stimulatory CAR truncated CD-19 expressing autologous TN/MEM cells	Phase 1	Intratumoral catheter
NCT05241392	Recruiting	B7-H3 targeting CAR-T cells	Phase 1	Ommaya device
NCT04077866	Recruiting	B7-H3 CAR-T + TMZ	Phase 1/2	ICT/ICV injection (B7-H3 CAR-T) + Oral (TMZ)
NCT05366179	Recruiting	CAR B7-H3T cells	Phase 1	ICV
NCT05474378	Recruiting	B7-H3CART	Phase 1	ICV or dual ICV/ICT
NCT04214392	Recruiting	Chlorotoxin (EQ)-CD28-CD3zeta-CD19t-expressing CAR T-lymphocyte	Phase 1	ICT or dual ICT/ICV
NCT03389230	Active, not recruiting	HER2(EQ)BBZ/CD19 + T cells	Phase 1	Intratumoral/intracavitary
NCT05540873	Recruiting	IL13alpha2 CAR-T cells	Phase 1	Infusion
NCT04003649	Recruiting	IL13Ralpha2-specific Hinge-optimized 41BBco-stimulatory CAR/Truncated CD19 expressing autologous TN/MEM+Ipilimumab+nivolumab	Phase 1	ICV (IL13Ralpha2 CAR T) IV (ipilimumab+nivoluma)
NCT05627323	Recruiting	CHM-1101 CAR-T cells	Phase 1	ICT/ICV dual delivery
NCT05353530	Recruiting	Ex-vivo expanded autologous IL-8 receptor (CXCR2) modified CD70 CAR (8R70CAR) T cells	Phase 1	Infusion
NCT05577091	Not yet recruiting	Autologous Tris-CAR-T cells	Phase 1	Infusion
NCT05168423	Recruiting	CART-EGFR-IL13Rα2	Phase 1	ICV
NCT05660369	Recruiting	CARv3-TEAM-E T cells	Phase 1	ICV

ICV = intracerebroventricular; ICT = intracranial intratumoral or intracavitary.

**Table 5:** Ongoing NK cell therapy trials in recurrent glioblastoma.

Trial Identifier	Status	Agent/Modality	Study Phase	Treatment Approach
NCT03383978	Recruiting	NK-92/5.28z + Ezabenlimab	Phase 1	ICT (NK-92/5.28z), infusion (Ezabenlimab)
NCT04254419	Not yet recruiting	NK cells	Phase 1	Infusion
NCT02100891	Active, not recruiting	Donor NK cell	Phase 2	Infusion

ICT = intracranial intratumoral or intracavitary.

**Table 6:** Published OV trials in recurrent glioblastoma.

Trial	Virus	Study Phase	Treatment Approach	Outcomes
Todo et al., 2022 [171], UMIN000015995	G47∆	Phase 2	ICT	mOS 20.02 months; OS-1yr = 84.2%
Ling et al., 2023 [173], NCT03152318	CAN-3110	Phase 1	ICT	mOS = 11.6 months
Markert et al., 2014 [174], NCT00157703	G207	Phase 1	ICT	mOS = 7.5 months; no DLT
Markert et al., 2009 [175]	G207	Phase 1b	ICT	mOS = 6.6 months; 1 case of transient fever, delirium, hemiparesis
Friedman et al., 2021 [176], NCT02457845	G207	Phase 1	ICT	mOS = 12.2 months; radiographic, neuropathologica,l or clinical responses in 11/12 patients
Harrow et al., 2004 [177]	HSV1716	Phase 1	ICT	No DLT
Papanastassiou et al., 2002 [178]	HSV1716	Phase 1	ICT	No DLT
Rampling et al., 2000 [179]	HSV1716	Phase 1	ICT	No DLT
Nassiri [147], NCT02798406	DXN-2401 + Pembrolizumab	Phase 1/2	ICT (DXN-2401); infusion (Pembrolizumab)	No DLT; ORR = 10.4%; OS-1yr = 52.7%
Chiocca et al., 2004 [180]	ONYX-015	Phase 1	ICT	No DLT; mOS = 6.2 months; mPFS = 46 days
Lang et al., 2018 [181]	DNX-2401	Phase 1	Single ICT; ICT followed by resection and injection into resection cavity	mOS (single ICT) = 9.3 months; mOS (ICT + resection) = 13.0 months
Samson et al., 2018 [182]	Reolysin	Phase 1b	IV infusion of 10^10^ TCID_5_	Reovirus RNA extensively detected in tumor cells, increased levels of PD-1/PD-L1 in treated patients
Kicielinski et al., 2014 [183]	Reolysin	Phase 1	ICT	mOS = 4.6 months; no severe treatment related adverse events
Forsyth et al., 2008 [184]	Reolysin	Phase 1	ICT	mOS = 4.8 months
Geletneky et al., 2017 [185]	Parvoryx01	Phase 1/2	(1) ICT followed by resection and injection around resection cavity; (2) Infusions for 5 days prior to resection and injection around resection cavity.	mOS = 15.3 months
Desjardins et al., 2018 [186]	PVSRIPO	Phase 1	ICT	mOS = 12.5 months

ICT = intracranial intratumoral or intracavitary; mOS=median overall survival; OS-1yr = 1-year overall survival; DLT = dose-limiting toxicity; ORR = objective response rate; mPFS = median progression-free survival; TCID = 50% tissue culture infectious dose.

**Table 7:** Ongoing OV trials in recurrent glioblastoma.

Trial	Status	Virus	Study Phase	Treatment Approach
NCT04482933	Not yet recruiting	G207	Phase 2	ICT
NCT02062827	Active, not recruiting	M032	Phase 1	ICT
NCT03657576	Recruiting	C134	Phase 1	ICT
NCT03896568	Recruiting	Allogeneic bone marrow-derived human mesenchymal stem cells loaded with DNX-2401	Phase 1	Infusion
NCT03911388	Recruiting	G207	Phase 1	ICT (cerebellum)
NCT03043391	Active, not recruiting	PVSRIPO	Phase 1	CED
NCT01582516	Completed	DNX-2401	Phase 1/2	CED
NCT01956734	Completed	DNX-2401	Phase 1	ICT
NCT00390299	Completed	MV-CEA	Phase 1	ICT
NCT02986178	Active, not recruiting	PVSRIPO:	Phase 2	CED
NCT02197169	DNX-2401	DNX-2401 with IFN-γ	Phase 1	ICT

ICT = intracranial intratumoral or intracavitary; CED = convention-enhanced delivery.
